# Mitigating the impact of COVID-19 on children's surgery in Africa

**DOI:** 10.1136/bmjgh-2020-003016

**Published:** 2020-06-10

**Authors:** Dennis Mazingi, George Ihediwa, Kathryn Ford, Adesoji O Ademuyiwa, Kokila Lakhoo

**Affiliations:** 1 Department of Surgery, University of Zimbabwe College of Health Sciences, Harare, Zimbabwe; 2 Paediatric Surgery Unit, Department of Surgery, Lagos University Teaching Hospital, Surulere, Lagos, Nigeria; 3 Department of Specialist Neonatal And Paediatric Surgery, Great Ormond Street Hospital, London, UK; 4 Department of Population, Policy and Practice, Institute of Child Health, University College London, London, UK; 5 Department of Surgery, College of Medicine, University of Lagos, Lagos, Lagos, Nigeria; 6 Nuffield Department of Surgical Sciences, Oxford University Hospitals NHS Trust, Oxford, Oxfordshire, UK

**Keywords:** surgery, public health

## Introduction

An outbreak of the disease known as COVID-19, which originated in Wuhan in the Hubei province of China, has rapidly spread to all continents of the globe.^1^ First detected via local hospital surveillance systems as a ‘pneumonia of unknown aetiology’ in late December 2019,^2^ the disease has since been declared a public health emergency of international concern by the WHO and reached pandemic status.

It is uncertain what the eventual toll of the pandemic will be in Africa; however, there has been a suspicion that the looming pandemic may hit harder than it has the rest of the world.^3 4^ Africa has baseline weaknesses in healthcare resource allocation, and her fragile healthcare systems are particularly vulnerable to being overwhelmed by this illness.^5 6^ Available statistics, to date, however, seem to show that the pandemic has been slow to begin. As of 26 May, 115 346 cases and 3471 deaths have been reported across the whole African continent, constituting 2% of all cases in the globe.^7^ African nations have had an opportunity to prepare for the coming onslaught, learn from the experience in other countries^3^ and choose interventions that are tailor-made for the unique socioeconomic context.^6^

While old age has consistently been associated with a higher risk of poor outcome, children appear to have escaped the worst of the disease.^8^ In a recent series from the Chinese Center for Disease Control and Prevention, less than 1% of the 73 314 cases were children below 10 years of age.^9^ Children of all ages may be affected, but they typically manifest mild or asymptomatic disease.^10 11^ This has important implications for the African pandemic: sub-Saharan Africa is the youngest continent in the globe with 63% of its population below the age of 25 years.^12^ The demography of Africa appears to portend a favourable course through the pandemic; however, it is unknown how the high prevalence of HIV infection, tuberculosis, malnutrition and the scourge of poverty will affect the human impact of the disease.^13^

The COVID-19 pandemic has placed unprecedented strain on health services around the world, and paediatric surgical services are no exception.^14 15^ Responses from surgical societies around the world thus far have focused on maintaining provision of emergency and urgent elective services while protecting healthcare workers (HCWs).^13 16^ There is a risk of healthcare resources being diverted away from surgical care, potentially impeding progress towards global surgery goals for 2030.^17^ Paediatric surgical care may only be tangentially affected by this pandemic; however, there are unique considerations that deserve special attention. This article explores the wider implications for children's surgery in Africa, drawing lessons from the past and giving recommendations for the current pandemic and future (table 1).

**Table 1:** Recommendations for paediatric surgery during the COVID-19 pandemic based on the domains identified including justification

Domain	Recommendations	Justification
Surgical rationing	Consider continuing surgery for paediatric malignancies. Consider adding waiting list length to inform surgical rationing in addition to urgency classifications.	To minimise a precipitous drop in surgical volumes that the service may not recover from
Surgical provision	Consider surgical approaches that minimise length of hospital stay.	To minimise the chance of nosocomial spread
HCW welfare	HCW life insurance cover in the event of death or incapacitation.^19^ Psychological support.^13^ Frequent personal protective equipment training and retraining.	To protect HCWs
Guardian policy	Designate a ‘resident/in-hospital guardian’ who lives on hospital grounds and is isolated from outside visitors. Limit in-hospital guardians to one person to prevent overcrowding. Hand hygiene for in-hospital guardians.^49^	To facilitate social distancing and minimise the chance of nosocomial spread
Visitor policy	Restrict all non-essential visitors.^49^ Restrict visitors for suspected or confirmed cases.^49^ Restrict visitation by any ill individual or family member.^49^	To reduce the number of vectors and minimise the chance of nosocomial spread
Child protection	Designate areas separate from general wards for children who may require protection during the pandemic. Strengthen social service structures during pandemics.	To protect children
Training	Training programmes to introduce virtual didactics. Consider altering trainee minimum requirements in light of declines in surgical volume and learning time. Increased collaborative learning between training programmes across borders.	To minimise the disruption to paediatric surgery workforce growth To strengthen surgical training programs

HCW, healthcare worker.

## Rationing of surgical services

Non-essential surgical and non-surgical activities should be curtailed to provide surge capacity for the expected pandemic-related influx. This is consistent with guidelines from many surgical societies worldwide^13 16^; however, heavy-handed shutdown policies have been discouraged in the African context because they risk exacerbating the already formidable surgical disease burden with disastrous consequences.^13 18^ Elective surgical activity has been postponed in Zimbabwe,^19^ South Africa,^20^ Kenya^21^ and Malawi, among many other countries. Negative effects should be anticipated if the past is anything to go by. During the 2003 severe acute respiratory syndrome-related coronavirus (SARS-CoV)-1 outbreak in Toronto, stringent restrictions on non-essential surgical services were thought to have aggravated precipitous declines in surgical volume, with only small increases in surge capacity for the outbreak.^22^ Postpandemic waiting lists for paediatric cancer are also expected to be sizeable.^4^ A recent modelling study from the ‘COVIDSurg Collaborative’ paints a grim picture. Twenty-eight million surgical operations are estimated to be cancelled and low-income and middle-income countries (LMICs) such as Africa will be hardest hit.^23^ The expectation that surgical volumes will bounce back rapidly is implausible, particularly in countries where there was already baseline fragility, and it may take longer than the 45 weeks forecast to make up the backlog.^23^ Current surgical rationing policies are based on a classification of the urgency of the patient’s intervention, such as the National Confidential Enquiry into Patient Outcome and Death system.^19 24^

## Effects on surgical practice

Paediatric surgical services in Africa are characterised by significant delays in health-seeking and within the referral chain.^25^ The mobility restrictions imposed on patients by shelter-in-place measures, as well as reduced income during the pandemic, will presumably cause further delays in presentation that may adversely affect outcomes.

The change to non-operative treatment in eligible patients for certain conditions, for example, appendicitis that is being contemplated, may find less success in Africa, where a higher proportion of patients have complicated disease not amenable to non-operative treatment.^26^ It also has the potential to prolong hospital stay,^27^ which increases the chances of nosocomial transmission of the virus.

## Preoperative screening and testing

Perinatal transmission of SARS-CoV-2 has not yet been demonstrated in recent small case series and a systematic review.^28–30^ This is consistent with findings during the SARS-CoV-1^31^ and Middle East respiratory syndrome (MERS-CoV) epidemics^32^ and should reassure surgeons working with neonates. However, neonates can still acquire infection from an infected mother's respiratory secretions.^33^ Also, Xu *et al* reported on eight infants who tested positive on rectal swabs even after having tested negative by nasopharyngeal swabs.^34^ This was thought to potentially represent faeco-oral viral transmission and has implications for surgeons of the gastrointestinal tract. SARS-CoV-2 has also been isolated in peritoneal fluid.^35^ Larger studies are needed to determine the significance of these findings. Airborne and contact precautions are indicated in all HCWs working with children of all ages.

## Healthcare workers

Experience from previous pandemics has demonstrated that HCWs are the lynchpin of resilient surgical systems during an outbreak. During the Ebola outbreak, the unfortunate death of 25% of the surgeons in one institution has led to a 97% reduction in surgical volumes,^36^ while trepidation on the part of HCWs and lack of personal protective equipment have led to a reluctance to work during the SARS-CoV-1,^37^ MERS-CoV^38^ and Ebola^39^ outbreaks. This is particularly damaging in Africa, where HCW morale is already low.^40^ HCW should be first in the minds of policy-makers because the axiom that there is no health without a workforce is as true during a pandemic as it is at any other time.^41^

Children have been called ‘the link in the transmission chain’ because of their importance in facilitating and amplifying viral transmission.^42^ Paediatric care in Africa is typically characterised by significant involvement by guardians and other family members who support the child during hospital admission, assist the overburdened healthcare workforce and act as care advocates.^43 44^ They frequently live on the hospital grounds because of long distances from home and prohibitive transportation costs.^43^ A study from Malawi showed that overcrowding in the hospital was a major issue due to the large population of guardians in the hospital.^45^ This is at odds with social distancing policies and has the potential to accelerate nosocomial transmission. Guardians should be limited to the minimum practical number per patient (table 1). Guardian policy should also take into account ‘parental presence at induction of anaesthesia’, a common practice that facilitates administration of anaesthesia but potentially places the parent at risk during an aerosol-generating procedure.

Hospital visitors have been implicated as vectors in pathogen transmission during the SARS-CoV-1 outbreak of 2002–2004,^46 47^ and hospital visitor policies were changed accordingly.^48^ The evidence linking restrictive visiting policies with prevention of nosocomial transmission during outbreaks is scant; however, it is a rational approach until better evidence comes to light. Expert guidelines from the Society for Healthcare Epidemiology of America give recommendations for guardian and visitor policy based on a systematic review of the literature and are incorporated in our recommendations^49^ (table 1).

## Child protection during pandemics

Experiences from this and past epidemics show that in health emergencies children, the most vulnerable members of society suffer disproportionately.^50^ ^51 52^ The ‘Agenda for Action’ recently announced by UNICEF is a timely intervention aimed at preventing the pandemic from becoming a child’s-rights crisis.

The incidence of family violence^53^ and accidental household trauma, for example, burn injuries, are anticipated to rise during the pandemic and is associated with shelter-in-place measures.^54^ Paediatric surgeons have a unique role in management of the traumatic injuries, protection of children from a dangerous household and in tertiary prevention (minimising the effects of child physical abuse and preventing recurrence).^52 55 56^ Churches, schools and shelters, which would otherwise be safe havens, may be closed and healthcare facilities may be the option of last report. Bringing a child into a potentially hazardous hospital environment with the risks of nosocomial infection brings up difficult choices.

## Impact on training

Surgical training programmes are an additional casualty of the social distancing measures and surgical rationing. The reduction in elective surgical cases and clinics, as well as contact between teachers and trainees, has brought challenges in the delivery of surgical education worldwide.^57–59^ Academic training programmes have had to adapt rapidly to maintain the integrity of training programmes, ensure trainee welfare and comply with local laws. Postgraduate qualifying examinations of the West^60^ and South African^61^ colleges of surgeons scheduled for April and July, respectively, have been postponed; however, the examination of the College of Surgeons of East, Central and Southern Africa (COSECSA) scheduled for November have not yet been impacted.

A recent global review of paediatric surgical workforce density showed that a minimum of four paediatric surgeons per million children under 15 years of age would be required to achieve a survival of >80% for a group of four bellwether paediatric surgical conditions.^62^ This translates to a deficit of 6967 additional paediatric surgeons in LMICs required to attend to the almost 1742 billion children living there.^62^ The paediatric surgical workforce deficit in Africa is particularly large,^63^ and disruption of training programmes is likely to significantly affect achievement of workforce goals.

The pandemic has also presented opportunities for surgical education. Virtual didactics are poised to increase the size of the classroom and to allow easier collaborative learning between teams in different hospitals or countries. This is occurring all over the continent and the practice may persist long after the pandemic is over.

## Conclusions

The inexorable spread of COVID-19 around the world continues unabated and threatens to affect every clinical specialty. Children have unique needs and suffer disproportionately during health emergencies and therefore require enhanced protection. Paediatric surgeons in Africa have an important role during times such as these and should use tailor-made, context-appropriate strategies to minimise the impact on our patients and HCWs. Protection for HCWs should be the foremost in the minds of policy-makers as they are a precious and irreplaceable resource.
